# Prolactin Pro-Differentiation Pathway in Triple Negative Breast Cancer: Impact on Prognosis and Potential Therapy

**DOI:** 10.1038/srep30934

**Published:** 2016-08-02

**Authors:** Vanessa M. López-Ozuna, Ibrahim Y. Hachim, Mahmood Y. Hachim, Jean-Jacques Lebrun, Suhad Ali

**Affiliations:** 1 Department of Medicine, Cancer Research Program, McGill University Health Centre, McGill University, Montreal, Quebec, Canada; 2 Medical Microbiology Department, RAK Medical and Health Sciences University, UAE

## Abstract

Triple negative breast cancer (TNBC) is a heterogeneous disease associated with poor clinical outcome and lack of targeted therapy. Here we show that prolactin (PRL) and its signaling pathway serve as a sub-classifier and predictor of pro-differentiation therapy in TNBC. Using immunohistochemistry and various gene expression in silica analyses we observed that prolactin receptor (PRLR) protein and mRNA levels are down regulated in TNBC cases. In addition, examining correlation of PRLR gene expression with metagenes of TNBC subtypes (580 cases), we found that PRLR gene expression sub-classifies TNBC patients into a new subgroup (TNBC-PRLR) characterized by epithelial-luminal differentiation. Importantly, gene expression of PRL signaling pathway components individually (PRL, PRLR, Jak2 and Stat5a), or as a gene signature is able to predict TNBC patients with significantly better survival outcomes. As PRL hormone is a druggable target we determined the biological role of PRL in TNBC biology. Significantly, restoration/activation of PRL pathway in TNBC cells representative of mesenchymal or TNBC-PRLR subgroups led to induction of epithelial phenotype and suppression of tumorigenesis. Altogether, these results offer potential new modalities for TNBC stratification and development of personalized therapy based on PRL pathway activation.

Triple negative breast cancer (TNBC) is typified by lack of expression of estrogen receptor (ER), progesterone receptor (PR), and human epidermal growth factor receptor-2 (HER-2). This subtype of breast cancer is characterized by poor histological characteristics, high rate of recurrence, poor patient outcome and lack of targeted therapy^1^,^2^. Loss of cellular differentiation is a key feature of TNBC tumors that may contribute to its unfavorable/aggressive phenotype^3^,^4^. Therefore, better understanding of the molecular pathways involved in cellular differentiation may provide new opportunities for better patient’s stratification, prognosis and personalized therapy in this breast cancer subtype^5^,^6^.

Studies performed to understand the biology of TNBC revealed that it is a heterogeneous disease^7^. Based on gene expression analyses, TNBC has been categorised into six subgroups including basal-like (BL1 and BL2), mesenchymal (M), mesenchymal stem-like (MSL), immunomodulatory (IM) and luminal androgen receptor (LAR)^8^. The basal-like (BL1 and BL2) subtypes are highly enriched in gene expression patterns associated with proliferation-related genes as well as genes involved in DNA damage response. The mesenchymal (M and MSL) subtypes are enriched in gene expression patterns associated with epithelial-to-mesenchymal transition process^8^. The immunomodulatory subtype is characterized for gene ontologies of immune cell processes that include immune cell and cytokine signaling, antigen processing and presentation^9^,^10^. The LAR subgroup is typified for being enriched in genes related with androgen receptor (AR) signalling and has been associated with good prognosis within TNBC^11^,^12^. In addition to this classification of TNBC other studies have generated various gene signatures distinguishing molecular subsets (basal-like, mesenchymal-like (claudin-low) and luminal androgen receptor) as well as non-neoplastic cell populations (epithelial claudin-CD24 signature, stromal signature, markers of blood, adipocytes, angiogenesis and inflammatory signature)^13^. Due to this diversity in the histological and molecular features as well as limited availability of well-defined molecular targets, developing treatments against TNBC remains challenging.

Extensive studies both *in vitro* and *in vivo* highlighted PRL and its downstream Jak2/Stat5 signaling pathway as central to mammary gland development and terminal differentiation of the mammary epithelial cells^14^,^15^. On the other hand, the role of PRL in breast cancer development/progression is not fully elucidated. Previous studies suggested that PRL could lead to breast cancer development by functioning as a local growth factor through a PRL/PRLR autocrine loop^16^,^17^,^18^,^19^. Furthermore, studies using transgenic mice designed to overexpress PRL in mammary epithelial cells resulted in the development of mammary tumors^20^,^21^. As well, PRL and PRLR were found to play a permissive role in oncogene-induced mammary tumors^22^. PRL was also found to cooperate with loss of p53 to induce claudin-low mammary carcinomas^23^ and was associated with interfering with BRCA1 regulation of expression of the cell cycle inhibitor p21^23^. In addition, PRL and PRLR were recently implicated in breast cancer metastatic spread^24^,^25^. While the above studies highlight a role for PRL in promoting tumorigenesis, many recent studies, including ours, suggested a different role as a potential suppressor of breast carcinogenesis. Indeed, we have previously shown that PRL, through PRLR/Jak2 signaling suppresses epithelial-mesenchymal-transition (EMT) and reduces the invasive properties of breast cancer cells^26^. Furthermore, using both mammary epithelial cells and human breast cancer cells we showed that PRL blocks growth factor-induced mammary cell proliferation and viability of breast cancer cells^27^. More recently we also found that expression of PRLR and PRL in human breast cancer correlate with favorable prognosis and better patient outcome^28^,^29^. In support of these findings, PRL and PRLR expression were found to be down regulated in breast cancer patients and breast cancer cell lines^30^,^31^. Moreover, expression/activation of the PRL effector molecule Stat5a was found to associate positively with increased levels of histologic differentiation of breast cancer tissues and to distinguish breast cancer patients with favourable prognosis and response to endocrine therapy^32^. Stat5a loss of expression was also found to be associated with tumor progression and unfavorable clinical outcomes^33^. As well, the PRL-responsive milk proteins whey acidic protein (WAP) and α-casein were also shown to inhibit tumorigenesis and breast cancer cell invasion^34^,^35^,^36^. Together these findings provide compelling evidence regarding the role of PRL pathway in maintaining tissue differentiation and as a suppressor of breast carcinogenesis. This unexpected suppressive role of PRL in breast cancer is still emerging and needs to be further elaborated. In addition, the role of PRL in TNBC has not yet been investigated.

In this study, we evaluated the role PRL differentiation pathway in prognosis and suppression of tumorigenesis in TNBC. Using tissue microarrays and gene profiling databases of breast cancer patients, our results identified a novel and relevant subgroup within TNBC characterized by PRLR expression and luminal-epithelial characteristics. This TNBC-PRLR subgroup showed better prognosis represented as prolonged disease free survival. Furthermore, functional studies using TNBC cell lines showed that activation of PRL signalling pathway suppresses the aggressive nature of TNBC cells *in vitro* and tumorigenesis *in vivo*. Overall, these findings propose a new management strategy for TNBC patients. This approach is based on screening for PRLR expression in patients that may benefit from the use of PRL hormone as a novel pro-differentiation therapy.

## Results

### PRLR expression is down regulated in TNBC

**Figure 1:**
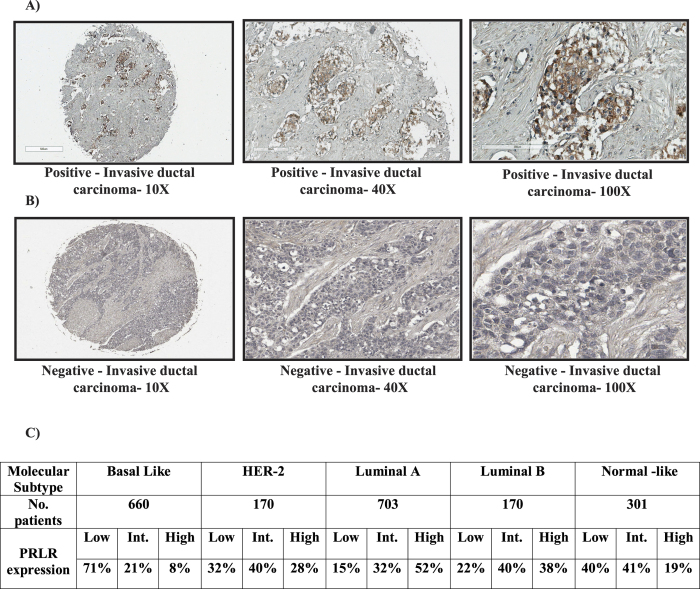
PRLR protein and gene expression in TNBC cases. (**A**) Positive immunohistochemical staining of PRLR in a case of invasive ductal carcinoma (10X, 40X and 100X). (**B**) Negative immunohistochemical staining of PRLR in a case of invasive ductal carcinoma (10X, 40X and 100X). (**C**) Table represents PRLR gene expression levels in different breast cancer molecular subtypes stratified according to robust single sample predictor classification (RSSPC) method using breast cancer gene-expression miner v3.0 database. PRLR gene expression levels were stratified into high, intermediate and low levels (Materials and Methods).

To decipher the role of PRL in TNBC, we first examined PRLR protein expression in TMA composed of 43 TNBC cases representing different grades, stages and histological types (Figure S1A). Interestingly, our analysis revealed that PRLR protein is expressed in only ~2% of the cases examined (Fig. 1A,B and Figure S1A). This down regulation of PRLR in TNBC cases was irrespective of grade, stage and histological type (Figure S1B–D). We next analyzed PRLR gene expression levels in different breast cancer molecular subtypes including TNBC (660 patients), Her-2 (170 patients), luminal A (703 patients) and luminal B (170 patients) using robust single sample predictor classification (RSSPC) in bc-GenExMiner 3.0 database. The Molecular subtype prognostic analysis tool of this program allows automatic beforehand classification of PRLR gene expression levels into three equal quartiles (low, intermediate and high). Our analyses showed that intermediate/high PRLR gene expression levels are least in TNBC (29%) compared to Her-2 (68%), luminal A (84%) and luminal B (78%) molecular subtypes (Fig. 1C). Together, these results indicate that while PRLR protein expression is down regulated in TNBC its gene expression is still preserved in 29% of cases.

### The prognostic relevance of PRL differentiation pathway in TNBC

**Figure 2:**
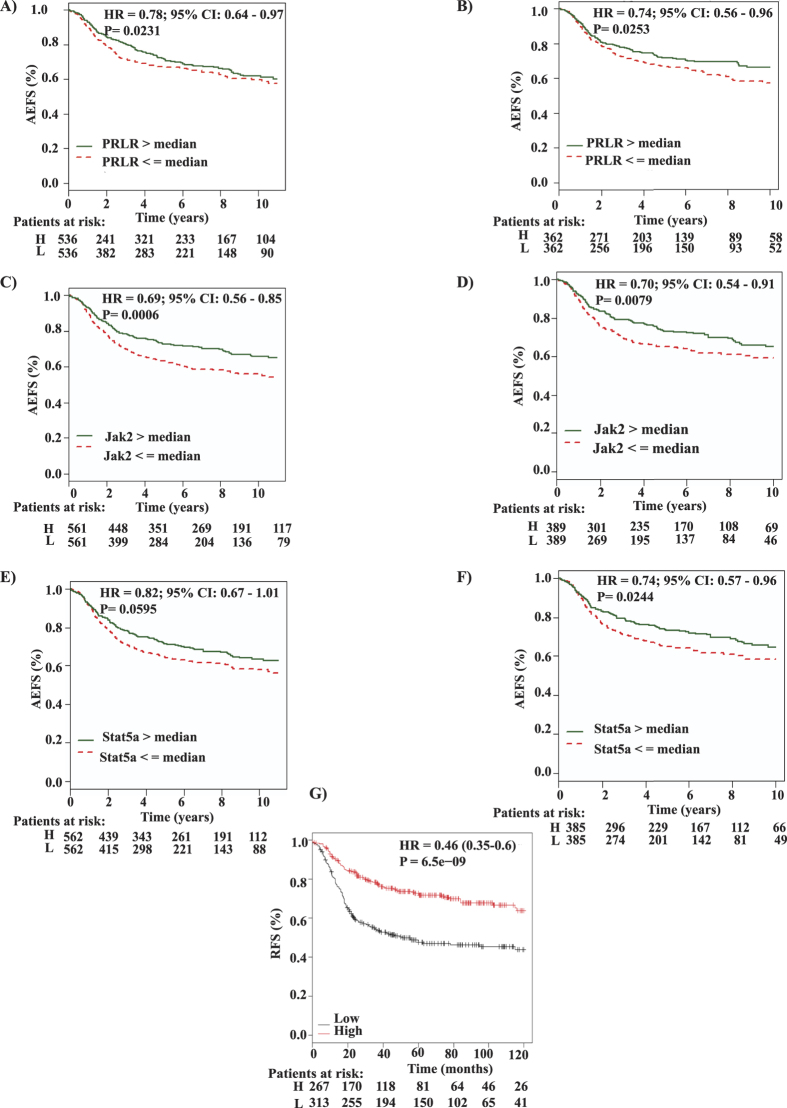
Expression of PRL signalling pathway components correlates with favorable patient’s outcome in TNBC. Kaplan-Meier survival curves for PRLR (**A,B**), Jak2 (**C,D**) and Stat5a (**E,F**) gene expression levels according to Hu *et al*. and Sorlie methods respectively using AEFS as an endpoint. Gene expression is stratified by median into high (green line) and low (red line) expression levels using breast cancer gene-expression miner v3.0. **G**) Kaplan-Meier survival curves for PRL pathway (PRL, PRLR, Jak2 and Stat5) based gene signature using RFS as an endpoint in basal breast cancer subtype using the KM plotter database.

Next we investigated whether PRL pathway expression could impact the prognosis of TNBC patients. To assess this point, we analyzed the association between PRLR gene expression and patient outcome, any event free survival (AEFS) using bc-GenExMiner 3.0 database of basal-like intrinsic breast cancer subgroup (representing TNBC) in two sub-classification methods (Hu and Sorlie)^2^,^37^. Interestingly, we observed a significant association between PRLR gene expression and prolonged AEFS (Hu 1,072 patients and Sorlie 724 patients) (Fig. 2A,B). Next we investigated the prognostic value of PRL signaling components Jak2 and Stat5a using the same methods of classification mentioned above. Interestingly, we also observed a significant correlation between Jak2 (Hu 1,122 patients and Sorlie 778 patients) (Fig. 2C,D) and Stat5a (Hu 1,124 patients and Sorlie 770 patients) (Fig. 2E,F) gene expression and prolonged AEFS.

Moreover, we investigated PRLR, Jak2 and Stat5a gene expression individually in relation to relapse free survival (RFS) using KM plotter database of TNBC patients^38^. Our results showed the same trend of significant association between PRLR, Jak2 and Stat5a gene expression and prolonged RFS (Figure S2) in 580 TNBC patients. We next investigated the prognostic power of PRL hormone gene expression in TNBC. Indeed, while PRL gene expression showed only marginal significance with better AEFS using Hu (1,139 patients) and Sorlie (783 patients) methods of classification (bc-GenExMiner 3.0), it was significantly associated with prolonged RFS using KM plotter database (580 patients) (Figure S3). For better evaluation of the prognostic role of PRL and its signaling pathway in determining TNBC patient outcome, we next generated a gene signature representing PRL pathway including PRL, PRLR, Jak2 and Stat5a. Gene expression levels were classified into high or low according to the mean expression levels for each gene grouped together. This was achieved using the multi-gene classifier tool of KM plotter database (Materials and Methods) in 580 TNBC patients. Importantly, our results showed a significant association between high PRL pathway based gene signature and prolonged RFS (P = 6.5e–09) in TNBC patients (Fig. 2G). Together, these findings indicate that PRL pathway expressers constitute a subgroup within TNBC patients displaying favorable outcome and prolonged survival.

### PRLR is a novel sub-classifier of TNBC patients

Recent studies have indicated the heterogenic nature of TNBC that impacts treatment options and patient outcome^8^,^13^. Therefore, here we examined PRLR gene expression in relation to metagenes representative of the molecular heterogeneity of TNBC using robust molecular subtype predictor classification (RMSPC) in bc-GenExMiner 3.0 that includes a cohort of 580 TNBC patients.

**Figure 3:**
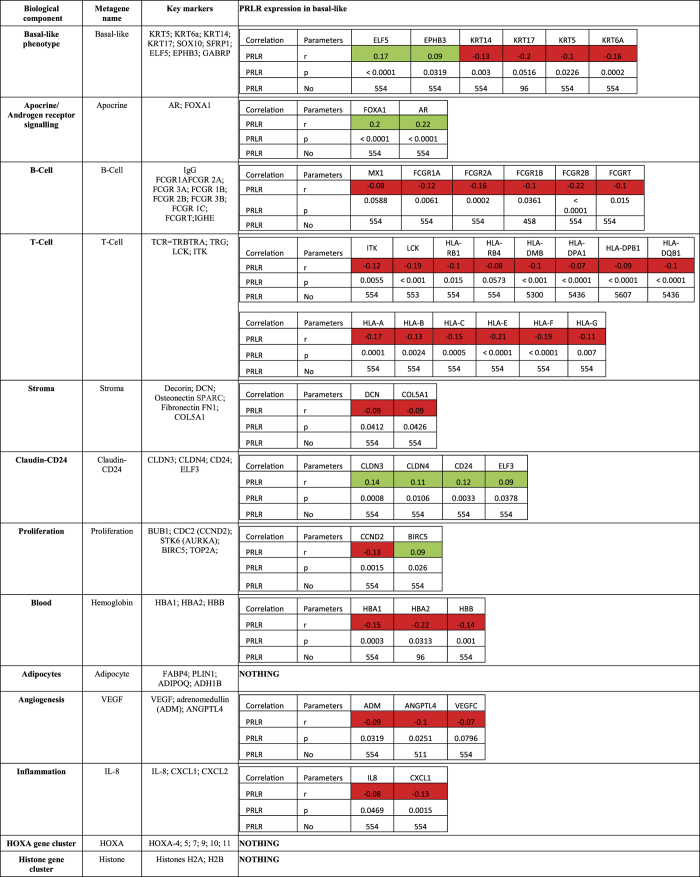
PRLR gene expression and its association with different TNBC gene signatures representative of molecular subtypes. Analysis of PRLR gene expression levels in association with different clusters of correlated genes (metagenes) used to distinguish molecular heterogeneity of TNBC. PRLR gene expression inversely correlates with genes related to basal-like subtype (basal keratins KRT14, KRT14, KRT5 and KRT6a), mesenchymal-like (claudin low) and genes representative of the non-neoplastic cell populations (red). PRLR expression shows a significant positive correlation with members of two metagene clusters (green) apocrine/androgen receptor signaling (FOXA1 and AR) and claudin-CD24 (CLDN3, CLDN4 and CD24) representing luminal/epithelial differentiation.

**Figure 4:**
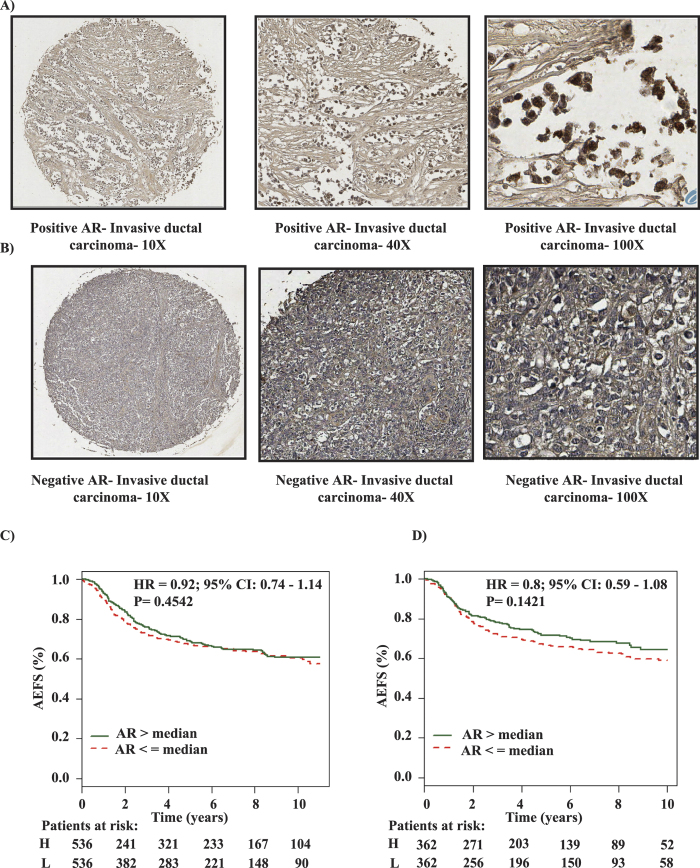
AR protein and gene expression in TNBC cases. (**A**) Positive immunohistochemical nuclear staining of AR in PRLR positive breast cancer case (4X, 40X and 100X). (**B**) Negative immunohistochemical staining of AR in a TNBC case (4X, 40X and 100X). (**C,D**) Kaplan-Meier survival curves for AR m-RNA levels in basal-like subtype stratified according to Sorlie’s and Hu’s classifications respectively, using AEFS as an endpoint using the breast cancer gene-expression miner v3.0. Gene expression is stratified according to median into high (green line) and low (red line) expression levels.

Interestingly, as shown in Fig. 3, PRLR gene expression was inversely correlated with genes related to basal-like subtype (basal keratins KRT14, KRT14, KRT5 and KRT6a), mesenchymal-like (claudin low) and genes representative of the non-neoplastic cell populations. However, PRLR gene expression showed a significant positive correlation with members of two metagene clusters. The first metagene represents luminal-like genes associated with LAR signaling (FOXA1 and AR) and the second metagene represents epithelial cell-cell adhesion and luminal differentiation (Claudin-CD24). These data suggest that PRLR identifies a novel and distinct subgroup of TNBC with luminal-epithelial differentiation. To validate and gain further insights into this new TNBC-PRLR subgroup, we analyzed the positive association between PRLR and AR protein expression using immunohistochemistry in the TMA of human TNBC cores used above^39^. As expected, AR protein expression was positive in ~29% of TNBC cases. Interestingly, only ~10% of these cases showed positive association with PRLR protein expression (Fig. 4A,B and Figure S4) confirming the metagenes association findings described above and highlighting that TNBC-PRLR is an independent subgroup. Previous reports have suggested that AR expression is a marker of favorable prognosis in TNBC^12^,^40^. Therefore, using the prognostic gene expression analysis tool of bc-GenExMiner 3.0 database, we then analyzed the prognostic value of AR gene expression in comparison to that of PRLR gene expression using the same methods of classification (Hu and Sorlie) as indicated in Fig. 2. In contrast to PRLR, AR gene expression showed no significant association with better patient outcome (Hu 1,072 patients and Sorlie 724 patients) (Fig. 4C,D). Together, these results indicate that PRLR expression is an independent biomarker of favorable patient outcome in TNBC and defines a novel TNBC subgroup.

### Restoring PRL signalling pathway in TNBC cells reduced cell viability, invasion capacity, mesenchymal properties and tumorigenesis

**Figure 5:**
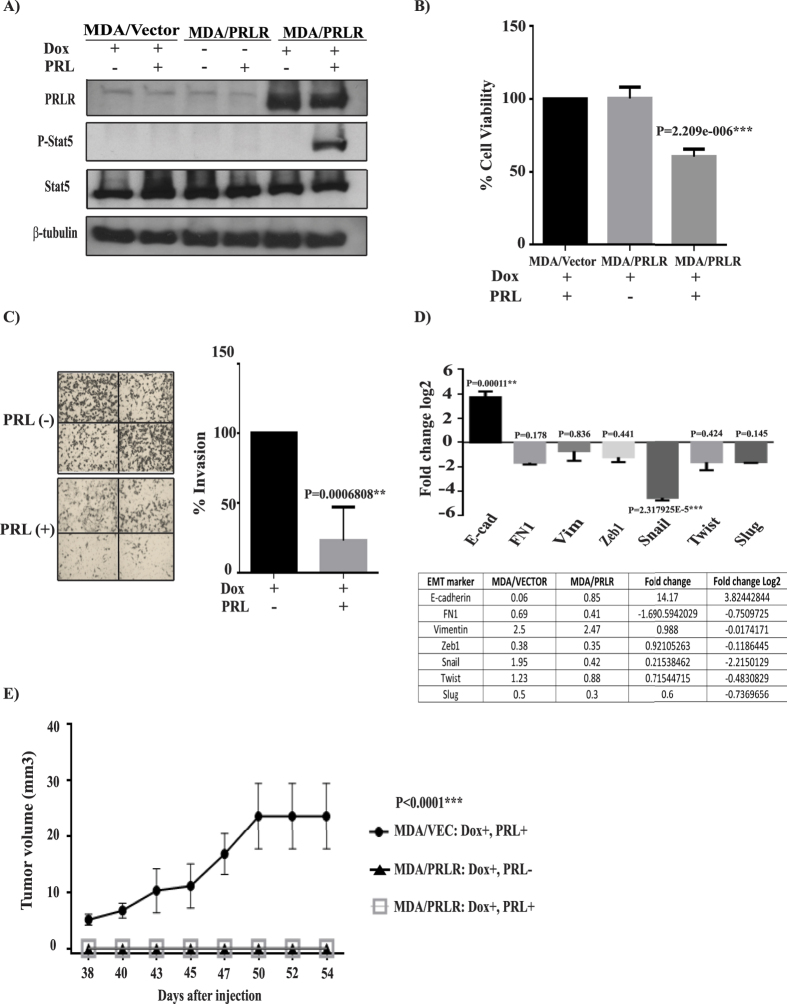
Restoring PRL-differentiation pathway in TNBC suppresses tumorigenesis. (**A**) Cells (MDA-MB-231/vector and MDA-MB-231/PRLR) were incubated in DMEM (2% FBS) and stimulated or not with dox (100 ng/ml) O/N and stimulated or not with hPRL 250 ng/ml for 15 minutes. Cell lysates were immunodetected using antibodies to PRLR, p-Stat5, Stat5 and β-tubulin. (**B**) Control MDA-MB-231/vector and MDA-MB-231/PRLR cells were plated in starvation media and treated or not with dox (100 ng/ml) and hPRL (250 ng/ml) for 72 hrs. MTT assays were performed and the results are presented as means ± SEM for triplicates of five independent experiments (p = 2.209e–006). (**C**) MDA-MB-231/PRLR cells were stimulated with dox (100 ng/ml) and stimulated or not with hPRL (250 ng/ml) for 72 hrs. Then equal number of cells was plated on Matrigel for invasion for 24 hrs. Columns represent means of triplicates of three independent experiments (P = 0.00068). Microscope images of invaded cells taken from 4 fields of a representative well (left). (**D**) MDA-MB-231/vector and MDA-MB-231/PRLR cells were treated with dox (100 ng/ml) and hPRL (250 ng/ml) for 72 hrs and the expression of EMT markers (as indicated) was examined using q-RT-PCR. Results are expressed as log2 fold change of triplicates of three independent experiments. (**E**) Graph depicting tumor volume of MDA-MB-231/Vector or MDA-MB-231/PRLR xenografts after treatment for a period of 8 weeks as indicated.

Previous studies have shown that the highly aggressive mesenchymal-like TNBC cell line MDA-MB-231 lacks expression of the PRLR^41^. To investigate the role of PRL and its signaling pathway in regulating TNBC biology we restored PRLR expression in MDA-MB-231 cell line using a dox-dependent lentiviral transduction method (Materials and Methods). As shown in Fig. 5A, significant PRLR protein expression was induced following dox treatment in MDA-MB-231/PRLR cells in comparison to MDA-MB-231/vector cells. In contrast to MDA-MB-231/vector cells, treatment of MDA-MB-231/PRLR cells with rhPRL led to the activation of PRL signaling molecule Stat5, indicative of a successful restoration of the PRL pathway in MDA-MB-231/PRLR cells (Fig. 5A). We next investigated the biological effects of restoring PRL signaling in regulating cell viability of MDA-MB-231 cells. Interestingly, our results showed that PRL treatment induced a significant decrease in cell viability (34–40%) of MDA-MB-231/PRLR cells in comparison to MDA-MB-231/Vector cells (P = 2.209e–006) (Fig. 5B). We next performed transwell invasion assays to gain further insights on the role of PRL pathway restoration in modifying the high invasive capacity of these TNBC cells. As shown in Fig. 5C, activation of PRL signaling pathway dramatically decreased the invasive capacity (78.5%) of MDA-MB-231/PRLR cells. As shown in Figure S5, this loss of invasive activity of MDA-MB-231/PRLR is not due to loss of cell viability. Next we examined the ability of PRL in regulating the expression of EMT markers including transcription factors (slug, snail, twist and zeb1) as well as E-cadherin, vimentin and fibronectin. As shown in Fig. 5D, we observed down regulation of all mesenchymal markers examined in MDA-MB-231/PRLR cells when compared to MDA-MB-231/vector cells following hPRL treatment. On the other hand, the epithelial marker E-cadherin was significantly up regulated by hPRL in MDA-MB-231/PRLR cells when compared to control MDA-MB-231/vector cells. Together these results indicate that restoring PRL pathway in TNBC suppress their aggressive behaviour and mesenchymal phenotype.

Finally, we analyzed the role of PRL pathway restoration in regulating tumorigenesis of MDA-MB-231 cells using NOD/SCID mouse xenograft animal model. Animals were inoculated with either MDA-MB-231/vector or MDA-MB-231/PRLR cells subcutaneously into the right flank of each mouse. Animals were randomly assigned into three groups: MDA-MB-231/vector and MDA-MB-231/PRLR treated with dox and hPRL and MDA-MB-231/PRLR treated with dox only. Animals were treated intra-peritoneal from day 1 following cell implantation and tumor growth was monitored for 8 weeks (Supplementary Materials and Methods). As shown in Fig. 5E, MDA-MB-231/vector xenografts showed considerable tumor growth reaching a volume of 20.81 mm^3^ at the time of sacrifice. Moreover, within the MDA-MB-231/PRLR untreated group only one mouse showed tumor growth that reached a maximum volume of 0.875 mm^3^ suggesting that mouse PRL while it is described as a weak agonist of the hPRLR, it is sufficient to induce activation of the overexpressed hPRLR. Importantly, all mice within the MDA-MB-231/PRLR treated group failed to develop any detectable tumors throughout the period examined (Fig. 5E) suggesting that PRL abrogates tumor formation *in vivo*. Altogether, these results indicate that restoration of the PRL pathway in TNBC results in suppression of cell viability, invasion capacity and tumorigenesis.

### PRL supresses cell viability and tumor growth of TNBC-PRLR subgroup

**Figure 6:**
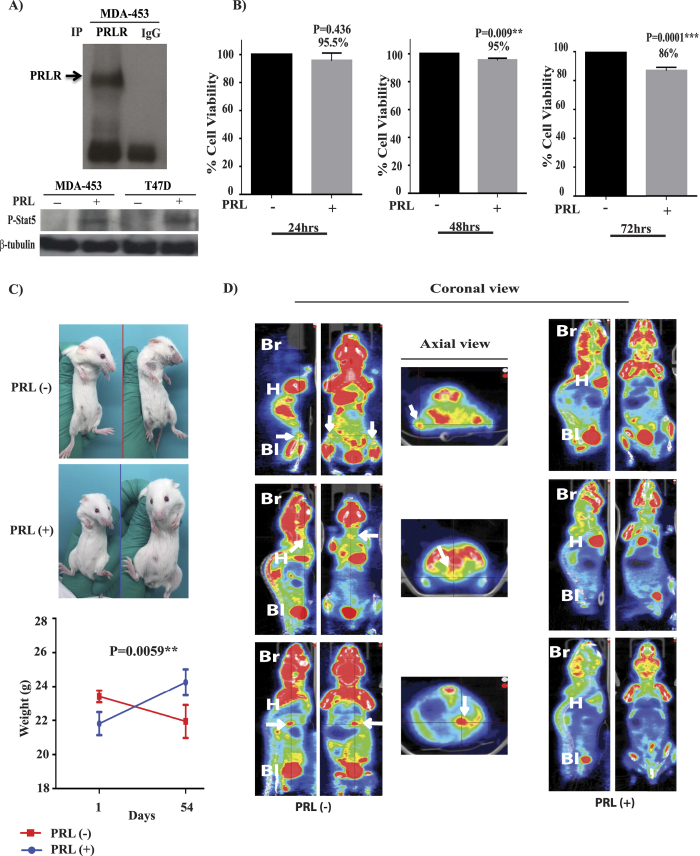
PRL suppresses viability and tumorigenesis of MDA-MB-453 cells representative of TNBC-PRLR subgroup. (**A**) MDA-MB-453 cells were lysed and immunoprecipitated using antibody to PRLR or control IgG and immunodetected using antibody to PRLR (Materials and Methods). MDA-MB-453 cells were incubated in L-15 (2% FBS) for an O/N period. Cells were then stimulated or not with hPRL (250 ng/ml) for 15 minutes. Cell lysates were immunodetected using antibodies to p-Stat5 and β-tubulin. (**B**) MDA-MB-453 cells (5 × 10^3^ cells) were plated in L-15 (2% FBS) and treated or not treated with hPRL (250 ng/ml) for 24–72 hrs as indicated. MTT assays were performed and results are presented as the mean ± SEM of triplicates of five independent experiments. (**C**) Representative pictures of NOD/SCID mice untreated or treated with r hPRL for 8 weeks. Graph depicting measurements of body weight in untreated or treated MDA-MB-453 xenograft mice. (**D**) Whole-body imaging of MDA-MB-453 xenograft using PET/SPECT/CT scan. FDG uptake is observed in brain (Br), heart (H) and bladder (Bl) as well as in xenograft tumors (white arrows).

Our previous results showed that PRLR gene expression sub-classifies a distinct population of TNBC tumors enriched with luminal and epithelial differentiation gene signatures associated with favourable outcome. To investigate the role of PRL and its signalling pathway in this TNBC subtype we tested PRL pathway activation in a representative cell line (MDA-MB-453). As shown in Fig. 6A, MDA-MB-453 cells express endogenous levels of PRLR^8^,^42^. PRL stimulation of these cells also resulted in Stat5 activation suggesting the presence of a functional PRL pathway in this cellular model of TNBC-PRLR subgroup. Next we investigated the role of PRL in regulating cellular viability in this model. Interestingly, we found that PRL caused a significant reduction in cell viability after 72 hrs of treatment (~15%) (P = 0.0001) (Fig. 6B). To further characterize the role of PRL in TNBC-PRLR subgroup, we analyzed the effects of PRL in regulating tumorigenesis using NOD/SCID/MDA-MB-453 animal xenograft model. Animals were inoculated with MDA-MB-453 cells subcutaneously into the right flank of each mouse. The mice were randomly assigned into two groups according to PRL treatment into MDA-MB-453 untreated and MDA-MB-453 treated (Supplementary Materials and Methods). Tumor growth and/or progression of the disease were monitored up to 8 weeks after cell inoculation. Notably, our results revealed that in the absence of PRL, mice showed signs of dissemination of the disease as well as a high incidence of morbidity (50%). These were assessed and measured by the appearance of paraneoplastic conditions (cachexia, anorexia, dehydration, respiratory difficulties, loss of weight, changes in the texture and coloration of the fur, skin dryness and loss of vibrissae), predominance of lethargic behavior as well as the presence of palpable tumors (Fig. 6C). In contrast, none of the above mentioned features were seen in the PRL treated group. PET/SPECT/CT studies were next performed to further evaluate disease progression. PET/SPECT/CT fusion of coronal and axial views of untreated group revealed the presence of high fludeoxyglucose (FDG) uptake area in the flanks, thymus and liver in addition to the expected normal tissues (brain, heart, and bladder) that normally exhibit a high rate of FDG uptake. Importantly, in the PRL treated group no FDG uptake was observed except for brain, heart, and bladder suggesting absence of tumor formation in this group of mice (Fig. 6D and Figure S6). All animals were subjected to necropsy to confirm the presence or the absence of secondary tumors by gross examination after PET/SPECT/CT analysis. The lack of tumor growth and/or dissemination in the PRL treated group was also confirmed by histological examination of lung and liver tissues (Figure S7). These findings further demonstrate the growth inhibitory effects of PRL *in vitro* and *in vivo* in TNBC-PRLR subgroup and highlight the possible use of PRL as a novel therapeutic strategy in TNBC. Altogether, these results emphasize that PRLR expression can categorize a specific TNBC subgroup with epithelial-luminal differentiation and favorable prognosis. Furthermore, PRLR can be used as a predictive marker for the possible use of PRL as a pro-differentiation therapy in breast cancer.

## Discussion

TNBC represents an enormous clinical challenge due to its aggressive nature, heterogeneity and lack of targeted therapy^6^,^43^. Histologically, the majority of TNBC tumors are poorly differentiated and show high grade, a characteristic that promotes the aggressive phenotype resulting in poor overall survival^44^. Recent advances in the field have helped in characterizing 6 different TNBC intrinsic subgroups^8^. Still, identification of novel biomarkers in this breast cancer subtype is critically needed to help understand the biology of TNBC and the development of new tools for prognosis and therapy.

Loss of cellular differentiation is a common feature of TNBC tumors. In addition, TNBC tumor cells are thought to originate from a progenitor mammary stem cell population. Therefore, elucidating the role of mammary differentiation pathways in TNBC biology might provide novel approachs in advancing classification, prognosis and treatment. PRL hormone is known to play an important role in mammary gland development and terminal differentiation of mammary epithelial cells^14^. The role of PRL in breast cancer development/progression is not fully elucidated and further studies are clearly required to clarify its role. Previous work described PRL and its receptor to play a permissive role in the development of mammary tumors and metastasis^16^,^17^,^18^,^19^,^20^,^21^,^22^,^23^,^24^,^25^. However recent studies have not only questioned this role of PRL but highlighted that it can act as a suppressor of breast tumorigenesis^26^,^27^. In addition PRL and PRLR were found to be down regulated in breast cancer and their expression correlate with good prognostic and better patient outcome^28^,^29^,^30^,^31^. This is consistent with recent evidence showing that PRLR receptor antagonists did not show any anti-tumorigenic effects and therapeutic benefits in clinical trials^45^.

Here we examined the prognostic and therapeutic role of PRL and its signalling pathway in TNBC. Our results indicate that while PRLR expression is down regulated in TNBC in comparison to other breast cancer molecular subtypes, intermediate/high PRLR mRNA levels are still preserved in ~30% of TNBC cases. Based on metagene cluster analyses we identified a TNBC-PRLR subgroup. This subgroup represents a distinct TNBC subgroup characterized by luminal-like differentiation (FOXA1 and AR) and epithelial (claudin-CD24) gene signatures. Moreover, our gene expression/prognosis analyses revealed that TNBC-PRLR subgroup has better patient overall survival outcomes in comparison to all subgroups of TNBC. Interestingly, these results are in agreement with a recent study describing PRL pathway to be enriched in the TNBC LAR subtype^46^,^47^. Altogether these results indicate that PRLR represents an independent and precise marker to distinguish a unique TNBC subgroup with specific molecular and prognostic features.

Nowadays, cytotoxic chemotherapy remains the mainstay of treatment for patients with TNBC in spite of the increasing number of targeted therapies. Targets such as epidermal growth factor receptor (EGFR)^48^, vascular endothelial growth factor receptor (VEGFR)^49^, DNA repair molecules^50^, cell-cycle control and cell survival genes^51^,^52^ as well as inhibitors of AR^53^ have been utilized to develop treatment modalities against TNBC. Still these treatment approaches show limited benefits, mostly due to toxic effects, resistance and tumor relapse. Pro-differentiation based therapies have been recently proposed in the hope of developing less aggressive treatments against cancer based on the reprograming/reversing cancer cells into less aggressive benign phenotype^45^,^46^. Our present study supports the pro-differentiation concept as a mean to revert/suppress tumorigenesis. Indeed, we provide *in vitro* and *in vivo* evidence indicating that restoration and activation of the PRL differentiation program in TNBC results in reversal of the highly proliferative, invasive, mesenchymal and tumorigenic phenotype through induction of cell differentiation. This reprograming into more epithelial and non-invasive features may explain the better overall survival seen in TNBC-PRLR subgroup.

Together our findings highlight the relevance of using differentiation pathways in suppressing tumorigenesis. Indeed, cancer cells are known to be plastic in nature and can be reverted to a less aggressive phenotype under favorable stimulus. Therefore exploiting pro-differentiation pathways in cancer should be considered as a viable avenue for developing novel prognostic and therapeutic approaches.

## Material and Methods

All experimental protocols were done in accordance with McGill University Health Centre, McGill University guidelines and regulations.

### Generation of stable cell lines

MDA-MB-231 parental cells were used to generate stable cell lines (MDA-MB-231/vector and MDA-MB-231/PRLR) overexpressing the human long from PRLR cDNA using doxycycline (dox)–dependent lentiviral system.

### Tissue microarray

TNBC tissue microarray (43 cases with clinico-pathological data) was purchased from US Biomax.

### Immunohistochemistry

Slides were incubated with a rabbit polyclonal antibody to PRLR-L (Santa Cruz #sc-20992) as describe previously^28^ and with a rabbit polyclonal antibody to AR (Santa Cruz #CO215), using positive and negative controls for both (Figure S1A and Figure S4 respectively).

### Gene expression analysis

Breast Cancer Gene-Expression Miner Version 3.0 (bc-GenExMiner 3.0) database was used to evaluate the mRNA levels of PRLR in different molecular subtypes^54^. As indicated in bc-GenExMiner 3.0 the biological validation of this tool was extensively tested and approved for different genes as indicated “ http://bcgenex.centregauducheau.fr/BC-GEM/GEM_Aide.php#Data_Validation”.

The prognosis gene expression analysis tool of bc-GenExMiner 3.0 was used to assess the association between PRL, PRLR, Jak2 and Stat5a mRNA levels and patient outcome, using gene symbol. The gene expression correlation analysis tool of bc-GenExMiner 3.0 was used to study the correlation between PRLR mRNA levels and members of the different metagenes within TNBC using robust single sample predictor classification (RSSPC)^13^. Kaplan-Meier plotter database was used to evaluate PRL signaling pathway components individually or as a single gene signature, using the following probeset ID (Affimetrix): PRL (205445_at), PRLR (206346_at), Jak2 (205841_at) and Stat5a (203010_at) in relation to patient outcome^55^.

### Cell lysis, immunoprecipitations and western blotting

For whole cell lysates and immunoprecipitations, cells were lysed in lysis buffer as described previously^56^.

### Invasion assay

80 × 10^3^ cells were seeded in 24-well plates HTS multi-well insert system coated with Matrigel. Invasion assays were performed for 24 hours as described previously^26^.

### RNA extraction and qRT-PCR

Total RNA from MDA-MB-231/PRLR and MDA-MB-231/vector cells treated with hPRL for 72 hrs was isolated, reverse transcribed and used for PCR amplification. RT-qPCR of EMT markers (slug, snail, twist, FN1, vimentin, E-cadherin, zeb1) was performed.

### MTT assay

MTT assays were performed as previously described^57^.

### Animal models

All experimental animal work was performed in a specific-pathogen-free animal facility according to the guidelines and ethical regulations of the Research Institute McGill University Health Centre approved animal used protocol (#2014-7492) in accordance with Canadian Council of animal care guidelines.

### MDA-MB-231 xenograft

18 Female NOD/SCID mice were purchased from Charles River Laboratories (Saint-Constant, QC, Canada) and randomly assigned into three groups (n = 6 mice/group) according to PRL treatment: MDA-MB-231/vector, MDA-MB-231/PRLR untreated and MDA-MB-231/PRLR treated. The mice were injected intra-peritoneal with doxycycline (20 mg/kg) daily. Treated group was injected intra-peritoneal every second day with rhPRL (0.1 μg/g). Tumor growth was monitored up to 8 weeks after implantation.

### MDA-MB-453 xenograft

12 Female NOD/SCID mice were purchased from Charles River Laboratories (Saint-Constant, QC, Canada) and randomly assigned into two groups (n = 6 mice/group) according to rhPRL treatment: MDA-MB-453 untreated or MDA-MB-453 treated. The mice were treated intra-peritoneal with either vehicle or rhPRL (0.1 μg/g) each second day. Tumor growth was monitored up to 8 weeks after implantation.

### Whole-body imaging of NOD/SCID/xenograft mice using PET/SPECT/CT scan

PET/SPECT/CT scan was performed on three mice from each group of MDA-MB-453 animal xenograft. At the end of the experiment mice were sacrificed by CO2 asphyxiation and subjected to necropsy.

## Additional Information

**How to cite this article**: López-Ozuna, V. M. *et al*. Prolactin Pro-Differentiation Pathway in Triple Negative Breast Cancer: Impact on Prognosis and Potential Therapy. *Sci. Rep*. **6**, 30934; doi: 10.1038/srep30934 (2016).

## Supplementary Material

Supplementary Information

## References

[b1] Viale G. The current state of breast cancer classification. Annals of oncology: official journal of the European Society for Medical Oncology/ESMO 23 Suppl 10, x207–210, doi: 10.1093/annonc/mds326 (2012). 22987963

[b2] Sorlie T. . Gene expression patterns of breast carcinomas distinguish tumor subclasses with clinical implications. Proceedings of the National Academy of Sciences of the United States of America 98, 10869–10874, doi: 10.1073/pnas.191367098 (2001). 11553815 PMC58566

[b3] Onitilo A. A., Engel J. M., Greenlee R. T. & Mukesh B. N. Breast cancer subtypes based on ER/PR and Her2 expression: comparison of clinicopathologic features and survival. Clinical medicine & research 7, 4–13, doi: 10.3121/cmr.2009.825 (2009). 19574486 PMC2705275

[b4] Nielsen T. O. . Immunohistochemical and clinical characterization of the basal-like subtype of invasive breast carcinoma. Clinical cancer research: an official journal of the American Association for Cancer Research 10, 5367–5374, doi: 10.1158/1078-0432.CCR-04-0220 (2004). 15328174

[b5] Perou C. M. . Molecular portraits of human breast tumours. Nature 406, 747–752, doi: 10.1038/35021093 (2000). 10963602

[b6] Foulkes W. D., Smith I. E. & Reis-Filho J. S. Triple-negative breast cancer. The New England journal of medicine 363, 1938–1948, doi: 10.1056/NEJMra1001389 (2010). 21067385

[b7] Abramson V. G., Lehmann B. D., Ballinger T. J. & Pietenpol J. A. Subtyping of triple-negative breast cancer: implications for therapy. Cancer 121, 8–16, doi: 10.1002/cncr.28914 (2015). 25043972 PMC4270831

[b8] Lehmann B. D. . Identification of human triple-negative breast cancer subtypes and preclinical models for selection of targeted therapies. The Journal of clinical investigation 121, 2750–2767, doi: 10.1172/JCI45014 (2011). 21633166 PMC3127435

[b9] Loi S. . Prognostic and predictive value of tumor-infiltrating lymphocytes in a phase III randomized adjuvant breast cancer trial in node-positive breast cancer comparing the addition of docetaxel to doxorubicin with doxorubicin-based chemotherapy: BIG 02-98. Journal of clinical oncology: official journal of the American Society of Clinical Oncology 31, 860–867, doi: 10.1200/JCO.2011.41.0902 (2013). 23341518

[b10] Denkert C. . Tumor-associated lymphocytes as an independent predictor of response to neoadjuvant chemotherapy in breast cancer. Journal of clinical oncology: official journal of the American Society of Clinical Oncology 28, 105–113, doi: 10.1200/JCO.2009.23.7370 (2010). 19917869

[b11] He J. . Prognostic value of androgen receptor expression in operable triple-negative breast cancer: a retrospective analysis based on a tissue microarray. Medical oncology 29, 406–410, doi: 10.1007/s12032-011-9832-0 (2012). 21264529

[b12] Tang D., Xu S., Zhang Q. & Zhao W. The expression and clinical significance of the androgen receptor and E-cadherin in triple-negative breast cancer. Medical oncology 29, 526–533, doi: 10.1007/s12032-011-9948-2 (2012). 21519872

[b13] Rody A. . A clinically relevant gene signature in triple negative and basal-like breast cancer. Breast cancer research: BCR 13, R97, doi: 10.1186/bcr3035 (2011). 21978456 PMC3262210

[b14] Hennighausen L. & Robinson G. W. Information networks in the mammary gland. Nature reviews. Molecular cell biology 6, 715–725, doi: 10.1038/nrm1714 (2005). 16231422

[b15] Ormandy C. J., Binart N. & Kelly P. A. Mammary gland development in prolactin receptor knockout mice. Journal of mammary gland biology and neoplasia 2, 355–364 (1997). 10935023 10.1023/a:1026395229025

[b16] Clevenger C. V., Furth P. A., Hankinson S. E. & Schuler L. A. The role of prolactin in mammary carcinoma. Endocrine reviews 24, 1–27, doi: 10.1210/er.2001-0036 (2003). 12588805 PMC1698952

[b17] Vonderhaar B. K. Prolactin involvement in breast cancer. Endocr Relat Cancer 6, 389–404 (1999). 10516853 10.1677/erc.0.0060389

[b18] Chen W. Y., Ramamoorthy P., Chen N., Sticca R. & Wagner T. E. A human prolactin antagonist, hPRL-G129R, inhibits breast cancer cell proliferation through induction of apoptosis [In Process Citation]. Clin Cancer Res 5, 3583–3593 (1999). 10589775

[b19] Chen N. Y. . *In vivo* studies of the anti-tumor effects of a human prolactin antagonist, hPRL-G129R. Int J Oncol 20, 813–818 (2002). 11894130 10.3892/ijo.20.4.813

[b20] Wennbo H. . Activation of the prolactin receptor but not the growth hormone receptor is important for induction of mammary tumors in transgenic mice. J Clin Invest 100, 2744–2751. (1997). 9389738 10.1172/JCI119820 PMC508478

[b21] Rose-Hellekant T. A. . Prolactin induces ERalpha-positive and ERalpha-negative mammary cancer in transgenic mice. Oncogene 22, 4664–4674 (2003). 12879011 10.1038/sj.onc.1206619 PMC1630768

[b22] Oakes S. R. . Loss of mammary epithelial prolactin receptor delays tumor formation by reducing cell proliferation in low-grade preinvasive lesions. Oncogene 26, 543–553 (2007). 16862169 10.1038/sj.onc.1209838

[b23] O’Leary K. A., Rugowski D. E., Sullivan R. & Schuler L. A. Prolactin cooperates with loss of p53 to promote claudin-low mammary carcinomas. Oncogene 33, 3075–3082, doi: 10.1038/onc.2013.278 (2014). 23873024 PMC4007359

[b24] Yonezawa T. . Anti-metastatic outcome of isoform-specific prolactin receptor targeting in breast cancer. Cancer Lett 366, 84–92, doi: 10.1016/j.canlet.2015.06.010 (2015). 26095602

[b25] Sutherland A. . The Role of Prolactin in Bone Metastasis and Breast Cancer Cell-Mediated Osteoclast Differentiation. J Natl Cancer Inst 108, doi: 10.1093/jnci/djv338 (2016). PMC5943829 26586670

[b26] Nouhi Z. . Defining the role of prolactin as an invasion suppressor hormone in breast cancer cells. Cancer Res 66, 1824–1832 (2006). 16452244 10.1158/0008-5472.CAN-05-2292

[b27] Haines E. . Tyrosine phosphorylation of Grb2: role in prolactin/epidermal growth factor cross talk in mammary epithelial cell growth and differentiation. Mol Cell Biol 29, 2505–2520, doi: 10.1128/MCB.00034-09 (2009). 19273609 PMC2682022

[b28] Hachim I. Y., Hachim M. Y., Lopez V. M., Lebrun J. J. & Ali S. Prolactin Receptor Expression is an Independent Favorable Prognostic Marker in Human Breast Cancer. Appl Immunohistochem Mol Morphol 24, 238–245, doi: 10.1097/PAI.0000000000000178 (2016). 26317306

[b29] Hachim I. Y., Shams A., Lebrun J. J. & Ali S. A Favorable Role of Prolactin in Human Breast Cancer Reveals Novel Pathway Based Gene Signatures Indicative of Tumor Differentiation and Favorable Patient Outcome: Prolactin-Induced Mammary Differentiation Program in Breast Cancer Prognosis. Hum Pathol, doi: 10.1016/j.humpath.2016.02.010 (2016). 26980025

[b30] Nitze L. M. . Reevaluation of the proposed autocrine proliferative function of prolactin in breast cancer. Breast cancer research and treatment 142, 31–44, doi: 10.1007/s10549-013-2731-7 (2013). 24146212 PMC3825490

[b31] Galsgaard E. D. . Re-evaluation of the prolactin receptor expression in human breast cancer. J Endocrinol 201, 115–128, doi: 10.1677/JOE-08-0479 (2009). 19153125

[b32] Yamashita H. . Stat5 expression predicts response to endocrine therapy and improves survival in estrogen receptor-positive breast cancer. Endocrine-related cancer 13, 885–893, doi: 10.1677/erc.1.01095 (2006). 16954437

[b33] Peck A. R. . Low levels of Stat5a protein in breast cancer are associated with tumor progression and unfavorable clinical outcomes. Breast cancer research: BCR 14, R130, doi: 10.1186/bcr3328 (2012). 23036105 PMC4053108

[b34] Bonuccelli G. . The milk protein alpha-casein functions as a tumor suppressor via activation of STAT1 signaling, effectively preventing breast cancer tumor growth and metastasis. Cell cycle 11, 3972–3982, doi: 10.4161/cc.22227 (2012). 23047602 PMC3507493

[b35] Nukumi N., Iwamori T., Kano K., Naito K. & Tojo H. Reduction of tumorigenesis and invasion of human breast cancer cells by whey acidic protein (WAP). Cancer letters 252, 65–74, doi: 10.1016/j.canlet.2006.12.005 (2007). 17215074

[b36] Ikeda K. . Inhibitory function of whey acidic protein in the cell-cycle progression of mouse mammary epithelial cells (EpH4/K6 cells). The Journal of reproduction and development 50, 87–96 (2004). 15007206 10.1262/jrd.50.87

[b37] Hu Z. . The molecular portraits of breast tumors are conserved across microarray platforms. BMC genomics 7, 96, doi: 10.1186/1471-2164-7-96 (2006). 16643655 PMC1468408

[b38] Zsuzsanna Mihály B. G. Improving Pathological Assessment of Breast Cancer by Employing Array-Based Transcriptome Analysis. Microarrays 2, 228–242 (2013). 10.3390/microarrays2030228 PMC5003464 27605190

[b39] Harvey J. M., Clark G. M., Osborne C. K. & Allred D. C. Estrogen receptor status by immunohistochemistry is superior to the ligand-binding assay for predicting response to adjuvant endocrine therapy in breast cancer. Journal of clinical oncology: official journal of the American Society of Clinical Oncology 17, 1474–1481 (1999). 10334533 10.1200/JCO.1999.17.5.1474

[b40] Luo X., Shi Y. X., Li Z. M. & Jiang W. Q. Expression and clinical significance of androgen receptor in triple negative breast cancer. Chinese journal of cancer 29, 585–590 (2010). 20507730 10.5732/cjc.009.10673

[b41] Ballestar E. . Methyl-CpG binding proteins identify novel sites of epigenetic inactivation in human cancer. The EMBO journal 22, 6335–6345, doi: 10.1093/emboj/cdg604 (2003). 14633992 PMC291845

[b42] Ormandy C. J., Clarke C. L., Kelly P. A. & Sutherland R. L. Androgen regulation of prolactin-receptor gene expression in MCF-7 and MDA-MB-453 human breast cancer cells. International journal of cancer 50, 777–782 (1992). 1544711 10.1002/ijc.2910500519

[b43] Dent R. . Pattern of metastatic spread in triple-negative breast cancer. Breast cancer research and treatment 115, 423–428, doi: 10.1007/s10549-008-0086-2 (2009). 18543098

[b44] Brouckaert O., Wildiers H., Floris G. & Neven P. Update on triple-negative breast cancer: prognosis and management strategies. International journal of women’s health 4, 511–520, doi: 10.2147/IJWH.S18541 (2012). PMC3469230 23071421

[b45] Agarwal N. . Phase I Study of the Prolactin Receptor Antagonist LFA102 in Metastatic Breast and Castration-Resistant Prostate Cancer. The oncologist, doi: 10.1634/theoncologist.2015-0502 (2016). PMC4861370 27091421

[b46] Yu K. D. . Identification of prognosis-relevant subgroups in patients with chemoresistant triple-negative breast cancer. Clinical cancer research: an official journal of the American Association for Cancer Research 19, 2723–2733, doi: 10.1158/1078-0432.CCR-12-2986 (2013). 23549873 PMC3655097

[b47] Burstein M. D. . Comprehensive genomic analysis identifies novel subtypes and targets of triple-negative breast cancer. Clinical cancer research: an official journal of the American Association for Cancer Research 21, 1688–1698, doi: 10.1158/1078-0432.CCR-14-0432 (2015). 25208879 PMC4362882

[b48] Baselga J. . Randomized phase II study of the anti-epidermal growth factor receptor monoclonal antibody cetuximab with cisplatin versus cisplatin alone in patients with metastatic triple-negative breast cancer. Journal of clinical oncology: official journal of the American Society of Clinical Oncology 31, 2586–2592, doi: 10.1200/JCO.2012.46.2408 (2013). 23733761 PMC5705191

[b49] von Minckwitz G. . Neoadjuvant chemotherapy and bevacizumab for HER2-negative breast cancer. The New England journal of medicine 366, 299–309, doi: 10.1056/NEJMoa1111065 (2012). 22276820

[b50] Farmer H. . Targeting the DNA repair defect in BRCA mutant cells as a therapeutic strategy. Nature 434, 917–921, doi: 10.1038/nature03445 (2005). 15829967

[b51] Lehmann B. D. & Pietenpol J. A. Targeting mutant p53 in human tumors. Journal of clinical oncology: official journal of the American Society of Clinical Oncology 30, 3648–3650, doi: 10.1200/JCO.2012.44.0412 (2012). 22965952

[b52] Lin J. . Targeting activated Akt with GDC-0068, a novel selective Akt inhibitor that is efficacious in multiple tumor models. Clinical cancer research: an official journal of the American Association for Cancer Research 19, 1760–1772, doi: 10.1158/1078-0432.CCR-12-3072 (2013). 23287563

[b53] Gucalp A. . Phase II trial of bicalutamide in patients with androgen receptor-positive, estrogen receptor-negative metastatic Breast Cancer. Clinical cancer research: an official journal of the American Association for Cancer Research 19, 5505–5512, doi: 10.1158/1078-0432.CCR-12-3327 (2013). 23965901 PMC4086643

[b54] Jezequel P. . bc-GenExMiner: an easy-to-use online platform for gene prognostic analyses in breast cancer. Breast cancer research and treatment 131, 765–775, doi: 10.1007/s10549-011-1457-7 (2012). 21452023

[b55] Gyorffy B. . An online survival analysis tool to rapidly assess the effect of 22,277 genes on breast cancer prognosis using microarray data of 1,809 patients. Breast Cancer Res Treat 123, 725–731, doi: 10.1007/s10549-009-0674-9 (2010). 20020197

[b56] Ali S. & Ali S. Prolactin receptor regulates Stat5 tyrosine phosphorylation and nuclear translocation by two separate pathways. The Journal of biological chemistry 273, 7709–7716 (1998). 9516478 10.1074/jbc.273.13.7709

[b57] Cocolakis E., Lemay S., Ali S. & Lebrun J. J. The p38 MAPK pathway is required for cell growth inhibition of human breast cancer cells in response to activin. The Journal of biological chemistry 276, 18430–18436, doi: 10.1074/jbc.M010768200 (2001). 11278744

