# Supplementary material for: Prolactin Pro-Differentiation Pathway in Triple Negative Breast Cancer: Impact on Prognosis and Potential Therapy
Source: Sci Rep. 2016 Aug 2;6:30934. doi: 10.1038/srep30934 (PMC4969612; doi:10.1038/srep30934)
Supplement: Supplementary Information [file srep30934-s1.pdf]

Prolactin Pro-Differentiation Pathway in Triple Negative Breast Cancer: Impact on Prognosis and Potential Therapy

Vanessa M. López-Ozuna[1], Ibrahim Y. Hachim[1], Mahmood Y. Hachim[2], Jean-Jacques Lebrun [3] and Suhad Ali[1*]

Supplementary Materials and Methods

All experimental protocols were done in accordance with McGill University Health Centre, McGill University guidelines and regulations.

Antibodies and reagents

Anti-PRLR antibody (polyclonal antibody) (H-300) against the intracellular domain of the PRLR long form (Santa Cruz #sc-20992) was used for IHC, western blotting and immunoprecipitation, Stat5 (invitrogen #13-3600), phospho-Stat5a (Invitrogen #71-6900) and β-tubulin (Santa Cruz #sc-53140) (monoclonal antibodies). Goat anti-mouse horseradish peroxidase (HRP) and goat anti-rabbit HRP purchased from Santa Cruz Biotechnology. Recombinant Human Prolactin (rhPRL) was purchased from Feldan Therapeutics (1F-02-008). According to manufacturer report the purity of the preparation is greater than 95% as determined by SDS-PAGE and endotoxin level is <0.01 EU per 1 μg of the protein by the LAL method (indicating the certainty that there is no possibility of the product to cause any bacterial contamination that can produce tissue injury or death).

Other reagents and materials include: doxycycline hyclate (Sigma #D9891-56), nitrocellulose membranes (Whatman), enhanced chemiluminescence hyperfilm and protein A-Sepharose beads (Amersham Biosciences/GE Healthcare), 24-well plates HTS multiwell insert system format (BD Falcon), 8.0μm pore size was used for invasion assays, 96-well plates (Corning #3753 and Fisher #7201216) were used for MTT assays.

Cell culture and generation of stable cell lines

MDA-MB-231 parental cells were used to generate stable cell lines overexpressing the human long from PRLR cDNA using doxycycline (dox)–dependent lentiviral system designated as

MDA-MB-231/vector and MDA-MB-231/PRLR according to manufacturer's instructions (Clontech) [26]. Cells were cultured in DMEM supplemented with 10% fetal bovine serum (Multicell, #095150). MDA-MB-453 cells obtained from Dr. Morag Park (McGill University) were cultured in L-15 supplemented with 10% fetal bovine serum (ATCC 30-2008).

Tissue microarray

Tissue microarray of TNBC cases (BR487) was commercially purchased from US Biomax (Rockville, MD, USA). The TMA includes 37 cases of invasive ductal carcinoma, 2 cases of intraductal carcinoma and 2 cases of medullary carcinoma, 1 case of infiltrating lobular carcinoma and 1 case of lobular ductal mixed carcinoma. The ER, PR and HER-2 status were provided for all the cases. These cases were negative for the three markers. Stage (TNM) and grade were also provided. Biomax assured the accuracy of all the histological and pathological features. For further assurance of the pathological diagnosis all the cases were reviewed by one of the co-authors (anatomical pathologist). For quality assurance, T47D cell line (positive control) and MDA-MB-231 (negative control) were used for IHC of PRLR (Figure S2) and AR (Figure S4).

Immunohistochemistry

Slides were baked for 30 minutes at 55C, followed by deparafinization and rehydration. Antigen retrieval was performed in sodium citrate 10mM, pH 6.0 buffers. Slides were incubated with a rabbit polyclonal antibody against PRLR-L (H300) (Santa Cruz #sc-20992) or a rabbit polyclonal antibody against AR (Santa Cruz #CO215), UltraVision LP Detection System HRP Polymer & DAP Plus Chromogen (Thermo Fisher Scientific, Fremont CA) was used for

detection. The TMA slides were scanned using Aperio XT slide scanner (Leica Biosystems). Membranous and/or granular cytoplasmic staining of PRLR in >10% of malignant cell was considered positive. AR immunostaining was assessed using Allred score [39]. Two investigators including one anatomical pathologist, blindly from the clinical data evaluated the slides independently. If there is any discordance, simultaneous examination was performed to solve the differences.

Human breast cancer gene profiling databases and in silica analyses

The Breast Cancer Gene-Expression Miner Version 3.0 (bc-GenExMiner 3.0) database[54] categorized patients according different methods of classification, based on variations in gene expression patterns derived from different cDNA microarrays analysis. Within this database two methods of classification (Hu and Sorlie) were used containing large number of TNBC patients[2,37] as indicated in results section. This database was used to evaluate PRLR gene expression levels (660 patients), prognosis (AEFS) using the two different sub-classification methods mentioned above (patient number for each gene tested is indicated in results and figures) and correlation of expression (Metagene analysis available in 580 patients out of the 660) (as described in results)[13]. In addition, the breast cancer Kaplan-Meier plotter database (668 TNBC patients) which identify patients based on 2013 StGallen criteria using the expression of HER2, ESR1 and MKI67[55], was used to evaluate PRL signaling pathway components individually, PRL (580 patients), PRLR (580 patients), Jak2 (580 patients) & Stat5a (580 patients), or as a gene signature (580 TNBC patients) in relation to patient outcome represented by RFS.

Cell lysis, immunoprecipitations and western blotting

For whole cell lysates and immunoprecipitations, cells were lysed in lysis buffer as described previously[56]. Immunoprecipitations were done for 3 hr at 4°C using a poly clonal antibody to PRLR and protein A-Sepharose beads. Proteins were run on SDS-PAGE and transferred to nitrocellulose membrane for western blotting analysis using the appropriate antibodies.

Invasion assay

Invasion assays were performed as described previously [26]. Cells were seeded in 24-well plates HTS multiwell insert system format (BD Falcon), 8.0µm pore size. Inserts were coated with Matrigel (BD biosciences) 1/10 dilution. 80 x10^3 cells were plate inside the inserts in 500µl with a 2% FBS growth medium. The bottom of the well was filled with 10% FBS growth medium. Cells were treated for 24-72 hours with rhPRL 250ng/ml and doxycycline (100 ng/ml), plated and left to invade for 24 hours. Invaded cells were stained with 1ml of 0.2% Crystal Violet (Fisher) for 1 hr. Images collection, processing and automated cell counting was then performed (McGill University Life Sciences Complex Advanced BioImaging Facility (ABIF)). Results are presented as means ± standard errors of the means (SEM) performed as triplicates of three independent experiments. Statistical significance was assessed using student's t test analysis.

RNA extraction and qRT-PCR

MDA-MB-231/PRLR and MDA-MB-231/vector cells grown to confluence then were pre-treated with doxycycline (10-100ng/ml) before stimulation. Before ligand stimulation, cells were starved in DMEM (2% FBS) and stimulated with rhPRL 250ng/ml for a period of 72 hrs. Cells were lysed in 500 µl of trizol. Total RNA was isolated as described by the manufacturer (Invitrogen Life Technologies, Burlington, Ontario, Canada). Samples were quantified by absorbance at 260

nm. Aliquots of 300 to 400 ng of total RNA were used for reverse transcription and PCR amplification in one step using Brilliant II SYBR green quantitative real- time PCR (qRT-PCR) Master Mix kit, 1-Step (Stratagene Amsterdam, Zui-doost, The Netherlands) according to the manufacturer's recommendation. RT-qPCR of EMT markers (slug, snail, twist, FN1, vimentin, e-cadherin, zeb1) was performed and the specificity of the primers was then tested by a dissociation program at 95°C for 1 min, with a ramp-down to 65°C and then a ramp-up to 95°C (at the instrument default rate of 0.2°C/s). Dissociation curve analysis was performed after the completed q-PCR. Data were obtained by slowly ramping up the temperatures of reaction solutions from 65 to 95°C. For EMT markers quantitative gene expression analysis was performed using the following primers: GAPDH forward CCTCAACTACATGGTTTAC, GAPDH reverse GGGATTTCCATTGATGAC, zeb1 forward GAAAGTGTTACAGATGCAG, zeb1 reverse TTCCTTTCCTGTGTCATC, snail forward GAAAAGGGACTGTGAGTA, snail reverse GAATAGTTCTGGGAGACA, slug forward CTGGTCAAGAAGCATTTC, slug reverse GGGGAAATAATCACTGTATG, E-cad forward ACATACACTCTCTTCTCTC, E-cad reverse GTCATTCTGATCGGTTAC, vimentin forward AACCTGAGGGAAACTAAT, vimentin reverse TTGATAACCTGTCCATCT, twist forward GGAGACCTAGATGTCATTGTT, twist reverse ACGCCCTGTTTCTTTGAA, FN1 forward TGTGGTTAGTGTCTATGC, FN1 reverse GCGATCAATGTTGGTTAC.

MTT assay

$2.5x10^3$ cells of MDA-MB-231/vector and MDA-MB-231/PRLR were seeded into 96-well plate overnight and grown in 2% FBS in DMEM media. Cells were treated with rhPRL 250ng/ml for a period of 24-72hrs. Then, cells were incubated with 3-(4,5-dimethyl-2-thiazolyl)-2,5-diphenyl-

2H-tetrazolium bromide (MTT) at 37°C for 2hrs as previously described[57]. Results are presented as means ± standard errors of the means (SEM) for triplicates of three separate experiments. Statistical significance was assessed using student's t test analysis. 5 x 10^3 cells of MDA-MB-453 were seeded into 96-well plate overnight and grown in 2%FBS in L-15 media. Cells were then starved in 2% FBS starvation media and either treated or not with hPRL 250ng/ml for a period of 24-72hrs following the same procedure as mentioned above. Results are presented as means ± standard errors of the means (SEM) performed as triplicates of five independent experiments. Statistical significance was assessed using student's t test analysis.

Statistical analysis:

Pearson correlation coefficient was used to evaluate the correlation between PRLR and different members of the metagenes used to distinguish molecular heterogeneity of TNBC. Correlation coefficient was denoted as (r). P value is also provided to evaluate the linear dependence between the two genes. Any event free survivals (AEFS), relapse free survival (RFS) curves in different databases were plotted using the Kaplan-Meier method. In vitro assays were all performed in triplicates of at least three independent experiments. Results were shown as means ± SEM. Student's t-test was used to evaluate the statistical significance. Statistical analyses were performed using GraphPad Prism software.

MDA-MB-231 xenograft animal models

Female NOD/SCID mice (18 in total) were purchased from Charles River Laboratories (Saint-Constant, QC, Canada), housed and maintained under specific pathogen-free conditions (RI-MUHC animal facility). The mice were randomly assigned into three groups (n=6 mice/group):

MDA-MB-231/vector (dox+rhPRL), MDA-MB-231/PRLR (dox+rhPRL) and MDA-MB-231/PRLR (dox). Cells (3×10^6/ml) were re-suspended in Matrigel and implanted subcutaneously into the right flank of each mouse. The mice were injected intra-peritoneal with doxycycline (20 mg/kg) daily. MDA-MB-231/PRLR treated mice were injected intra-peritoneal every second day with rhPRL (0.1µg/g). Tumor growth was monitored up to 8 weeks after implantation. Tumor volume was measured in two dimensions with a vernier caliper (Mitutoyo, Kawasaki, Japan) and calculated using the formula [length x width^2] /2. Mice were euthanized by cervical dislocation following 8 weeks of treatments. For measuring serum levels of injected rhPRL, mice were anesthetised and blood was collected by cardiac puncture. Levels of serum rhPRL were determined using radioimmunoassay at 2hrs and 4hrs post injection (Table S1).

MDA-MB-453 xenograft animal models

Female NOD/SCID mice (12 in total) were purchased from Charles River Laboratories (Saint-Constant, QC, Canada) housed and maintained under specific pathogen-free conditions (RI-MUHC animal facility). The mice were randomly assigned into two groups (n=6 mice/group): MDA-MB-453 untreated and MDA-MB-453 rhPRL treated. Cells (5×10^6/ml) were re-suspended in Matrigel and implanted subcutaneously into the right flank of each mouse. The mice were treated intra-peritoneal with either vehicle or hPRL (0.1µg/g) each second day. Tumor growth was monitored up to 8 weeks after implantation. PET/SPECT/CT scan was performed (please see below) on three mice from each group. At the end of the experiment mice were sacrificed by CO_2 asphyxiation and subjected to necropsy.

Whole-body imagines of NOD/SCID/xenograft mice with PET/SPECT/CT scan analysis

The animals were kept fasting for approximately 12hrs before undergoing scanning. They were anesthetized using 1 L/min of 2% isoflurane in 100% oxygen. After receiving the anesthesia, they were injected in the tail vein with the radiotracer (fludeoxyglucose [^{18}F]) (FDG). Following an appropriate uptake period of 45 minutes, animals were re-anesthetized (isoflurane, 5% induction, 1.5-2% maintenance throughout the scan), moved to the Mediso nanoScan and placed in the prone position on an animal bed covered with absorbent paper. Following completion of PET and/or SPECT scans; a CT scan was performed for anatomical localization and attenuation correction. Respiration rate and body temperature was continuously monitored and the temperature maintained at ~37°C throughout the study using a feedback-regulated warming system. Following completion of scanning, the animals were euthanized. Data acquired in list-mode format for 60min, full 3D sonograms with corrected efficiency, scattering, attenuation, count losses and decay were reconstructed using an iterative 3D dynamic raw-action maximum likehood algorithm (Drama). After PET scanning a CT scan was performed. Analysis of the PET/SPECT and the CT datasets were imported using the nonproprietary AMIDE software, version 0.8.2 (http://amide.sourceforge.net)[58]. The imaging system used was the nanoScan pre-clinical SPECT/CT/PET (Mediso medical imaging systems, Hungary). The imaging was performed by RI-MUHC Small Animal Imaging Labs (SAIL) Platform, Centre for Translational Biology, Research Institute of the McGill University Health Centre, Montreal, Canada.

Supplemental Legends to Figures and Tables

Figure S1: A) Left panel: PRLR protein expression in a TMA of 43 TNBC cases. Middle panel: Positive immunohistochemical staining of PRLR protein in T47D human breast cancer cells (positive control). Right panel: Negative immunohistochemical staining of PRLR protein in MDA-MB-231 human breast cancer cells (negative control). B) Negative immunohistochemical stain of PRLR in ductal carcinoma in situ (10X, 40X and100X). C) Negative immunohistochemical stain of PRLR in invasive lobular carcinoma (10X, 40X and100X). D) Negative immunohistochemical stain of PRLR in medullary carcinoma (10X, 40X and100X).

Figure S2: Kaplan-Meier survival curves for PRLR (A), Jak2 (B), Stat5a (C) gene expression levels in basal-like subtype, using RFS as an endpoint in KM plotter database stratified with median into high (black line) and low (red line) expression levels (P values and number of patients are indicated).

Figure S3: A & B) Kaplan-Meier survival curves for PRL gene expression levels in basal-like subtype according to Hu and Sorlie methods respectively, using AEFS as an endpoint stratified by median into high (green line) and low (red line) expression levels using bc-GenExMiner 3.0 database. In each graph HR and P values and number of patients are indicated. C) Kaplan-Meier survival curves for PRL gene expression using RFS as an endpoint in basal breast cancer subtype using the KM plotter database.

Figure S4: left panel: Positive immunohistochemical staining of AR protein in T47D cells (positive control). Right panel: Negative immunohistochemical staining of AR protein in MDA-MB-231 (negative control).

Figure S5: MDA-MB-231/vector and MDA-MB-231/PRLR cells were plated in starvation media and treated or not with dox (100 ng/ml) and hPRL (250 ng/ml) for 48hrs as indicated. MTT assays were performed and the results are presented as means ± SEM for triplicates of three independent experiments.

Figure S6: Another coronal cut of whole-body imaging of MDA-MB-453 xenograft treated group using PET/SPECT/CT scan. FDG uptake is observed in brain (Br), heart (H) and bladder (Bl) as expected.

Figure S7: A) H&E staining of primary breast cancer tumor obtained from the flanks of MDA-MB-453 untreated mice. B) Gross examination of untreated MDA-MB-453 xenograft mice showing tumor-bearing livers and thymus (white arrows represent secondary tumors). C) H&E staining of liver and lungs of hPRL treated MDA-MB-453 xenograft mice with no evidence of metastasis.

Table S1: Serum levels of hPRL in MDA-MB-231/PRLR xenograft animal mouse model

Serum levels hPRL detected by radioimmunoassay at 2hrs and 4hrs post intra-peritoneal injection of recombinant hPRL (0.1μg/g of body weight) in untreated and treated mice.

A)

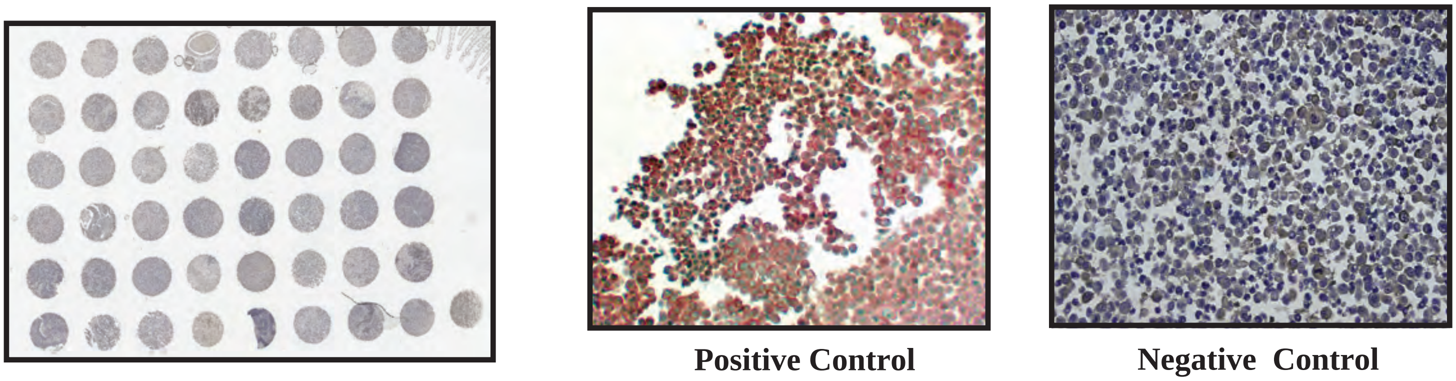

Positive Control (40X)

Negative Control (40X)

B)

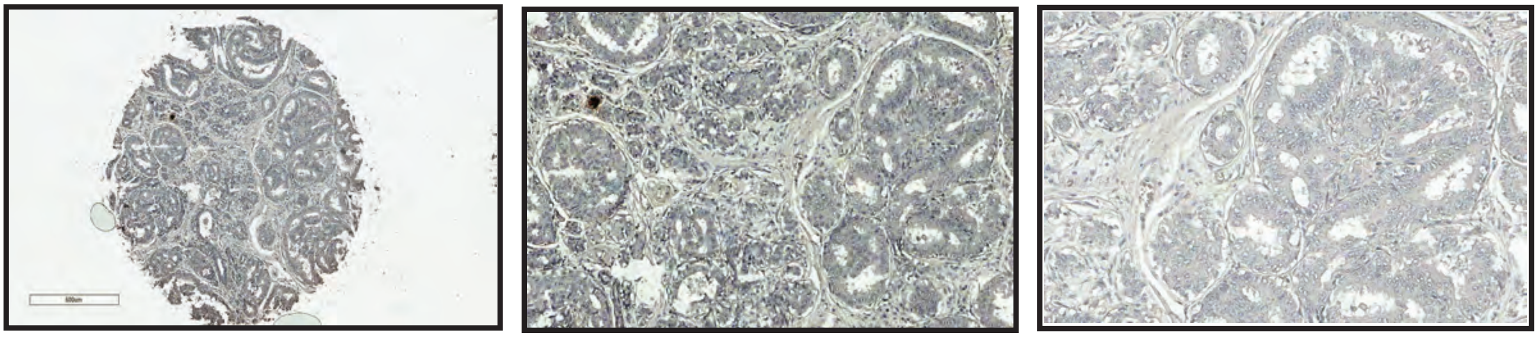

Negative - DCIS- 10X

Negative - DCIS- 40X

Negative - DCIS- 100X

C)

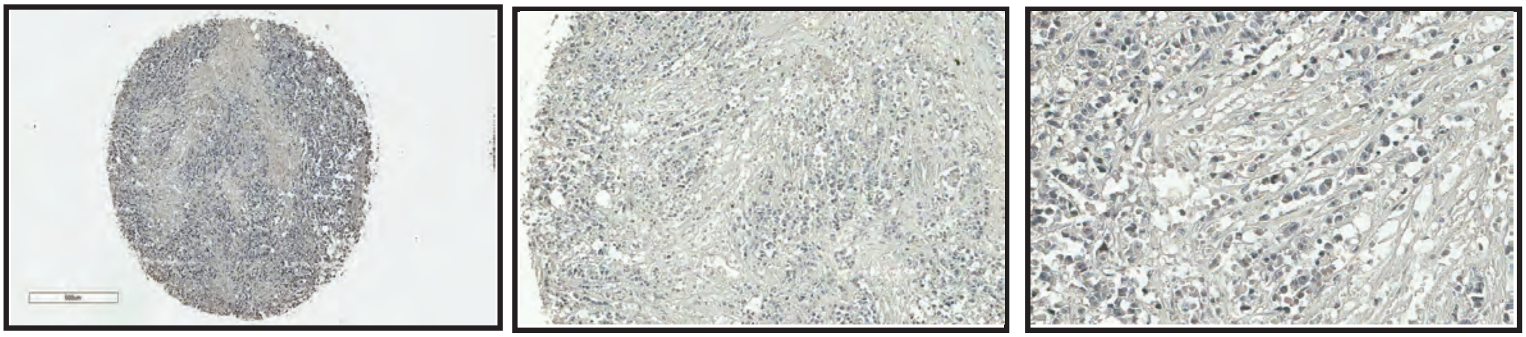

Negative - Invasive lobular carcinoma- 10X

Negative - Invasive lobular carcinoma- 40X

Negative - Invasive lobular carcinoma- 100X

D)

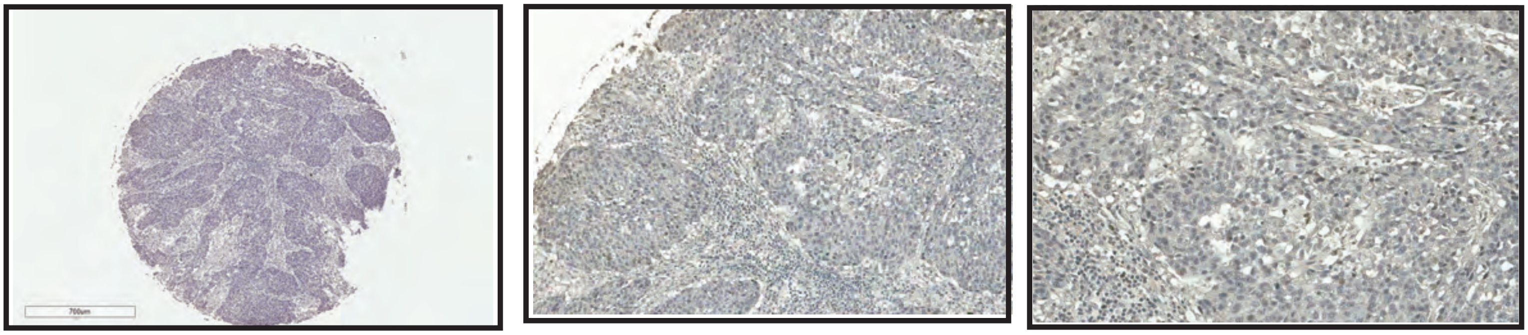

Negative- Medullary carcinoma - 10X

Negative- Medullary carcinoma - 40X

Negative- Medullary carcinoma - 100X

Figure S1

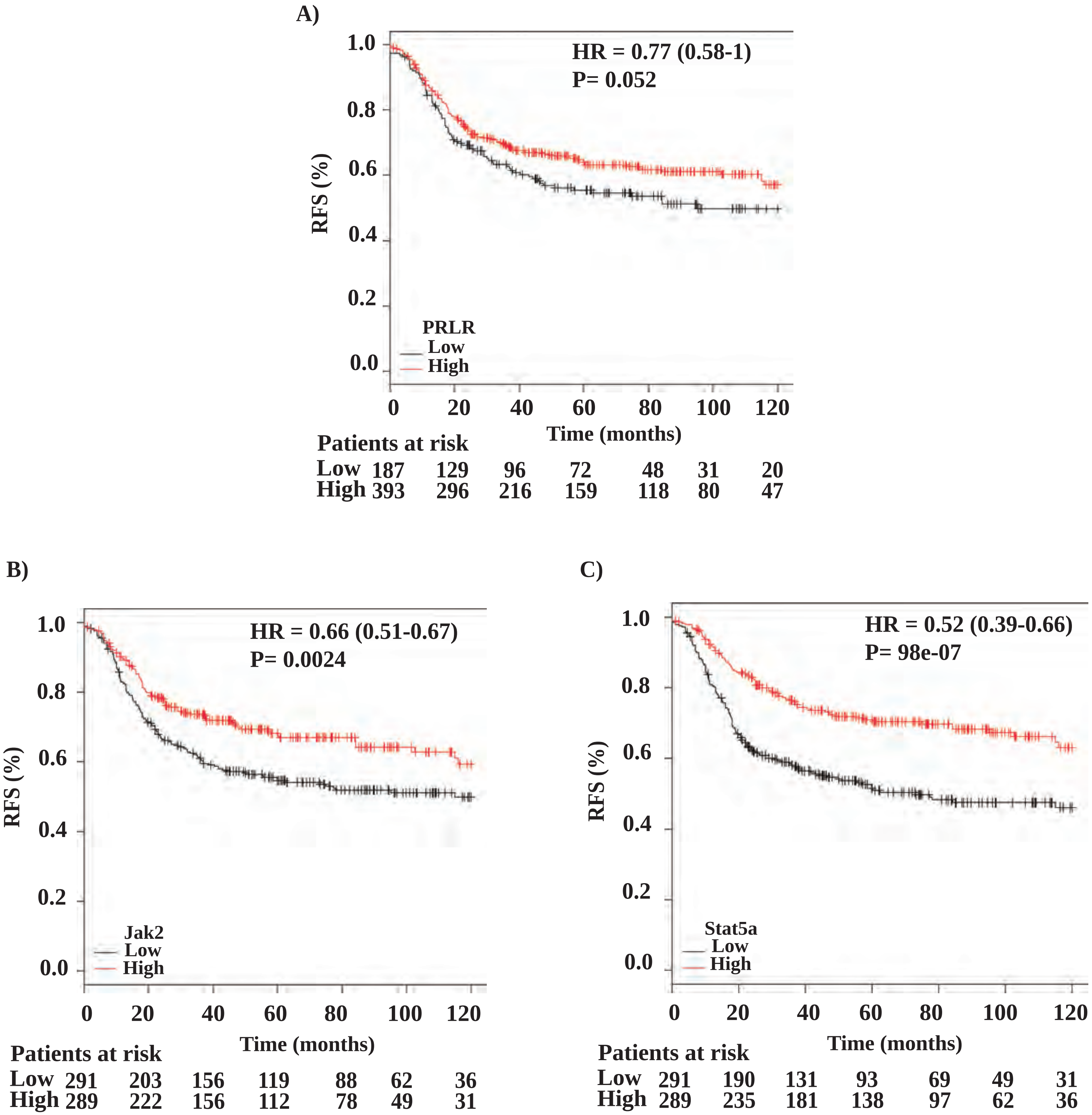
A)
HR = 0.77 (0.58-1)
P= 0.052
RFS (%)
1.0
0.8
0.6
0.4
0.2
0.0
PRLR
Low
High
0
20
40
60
80
100
120
Time (months)
Patients at risk
Low 187 129 96 72 48 31 20
High 393 296 216 159 118 80 47
B)
HR = 0.66 (0.51-0.67)
P= 0.0024
RFS (%)
1.0
0.8
0.6
0.4
0.2
0.0
Jak2
Low
High
0
20
40
60
80
100
120
Time (months)
Patients at risk
Low 291 203 156 119 88 62 36
High 289 222 156 112 78 49 31
C)
HR = 0.52 (0.39-0.66)
P= 98e-07
RFS (%)
1.0
0.8
0.6
0.4
0.2
0.0
Stat5a
Low
High
0
20
40
60
80
100
120
Time (months)
Patients at risk
Low 291 190 131 93 69 49 31
High 289 235 181 138 97 62 36

Figure S2

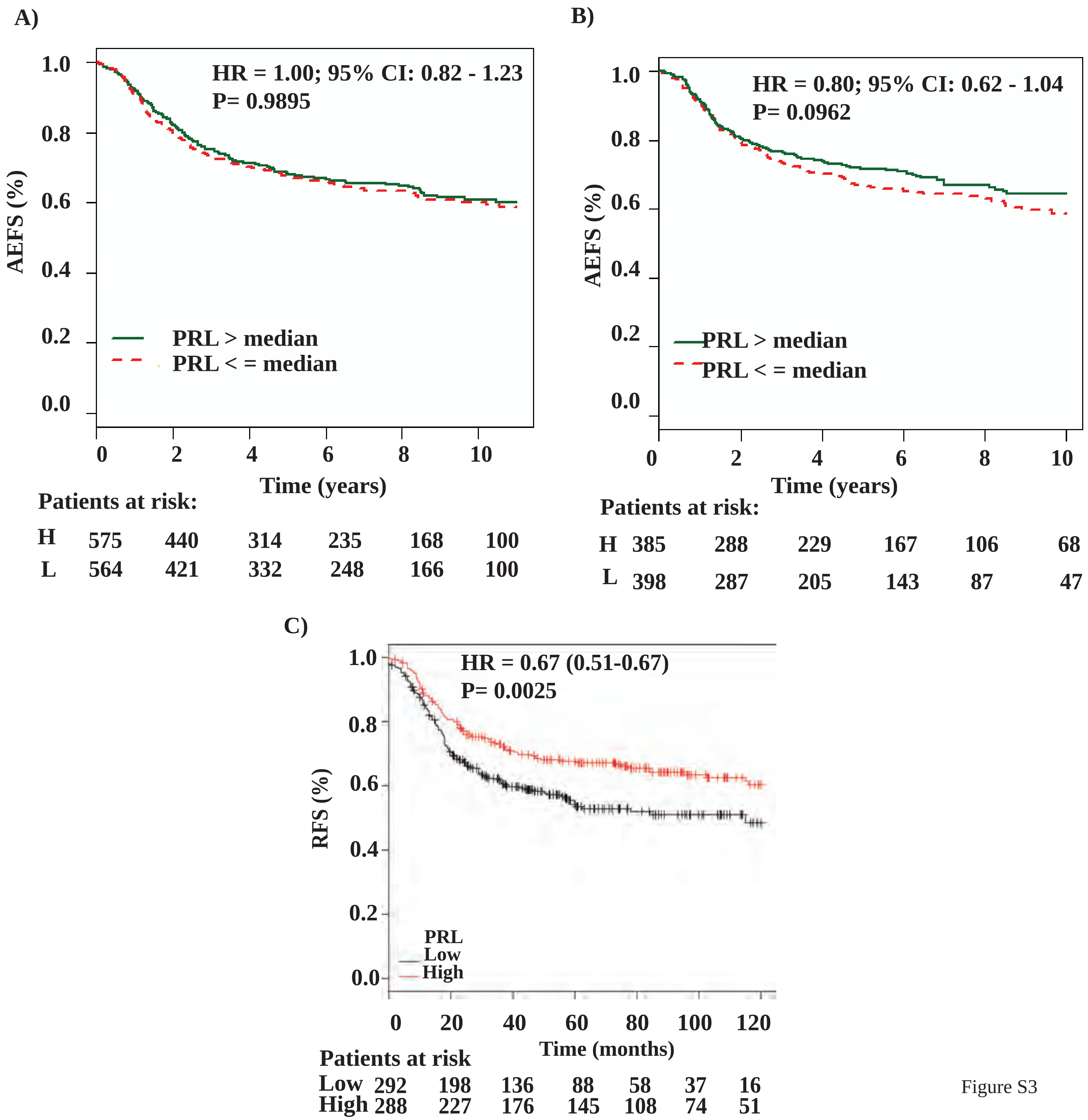
A)
HR = 1.00; 95% CI: 0.82 - 1.23
P= 0.9895
AEFS (%)
1.0
0.8
0.6
0.4
0.2
0.0
PRL > median
PRL < = median
0
2
4
6
8
10
Time (years)
Patients at risk:
H 575 440 314 235 168 100
L 564 421 332 248 166 100
B)
HR = 0.80; 95% CI: 0.62 - 1.04
P= 0.0962
AEFS (%)
1.0
0.8
0.6
0.4
0.2
0.0
PRL > median
PRL < = median
0
2
4
6
8
10
Time (years)
Patients at risk:
H 385 288 229 167 106 68
L 398 287 205 143 87 47
C)
HR = 0.67 (0.51-0.67)
P= 0.0025
RFS (%)
1.0
0.8
0.6
0.4
0.2
0.0
PRL
Low
High
0
20
40
60
80
100
120
Time (months)
Patients at risk
Low 292 198 136 88 58 37 16
High 288 227 176 145 108 74 51

Figure S3

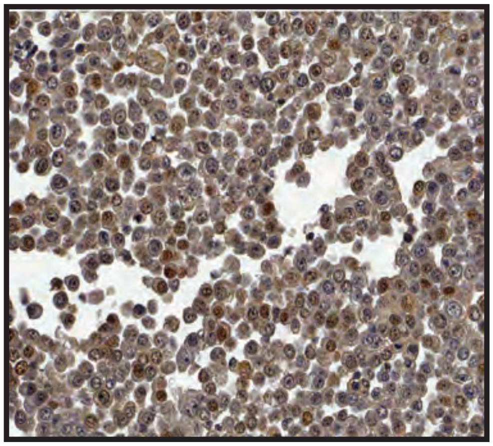

Positive Control
(40X)

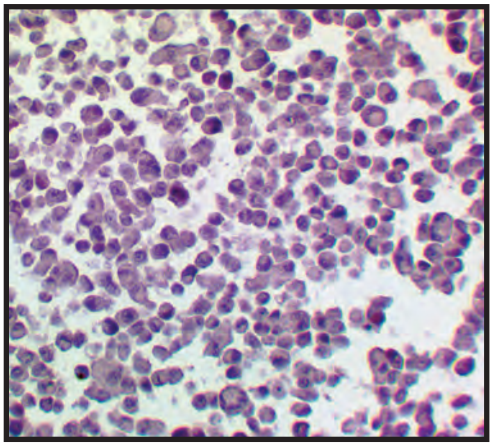

Negative Control
(40X)

Figure S4

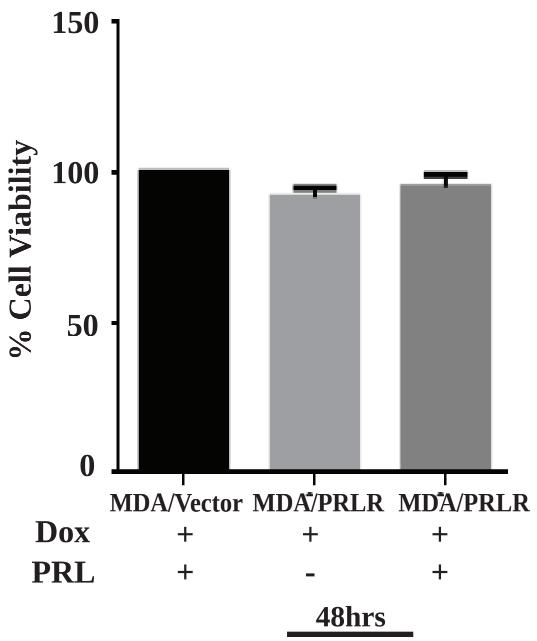

150
100
50
0
% Cell Viability
MDA/Vector
MDA/PRLR
MDA/PRLR
Dox
+
+
+
PRL
+
-
+
48hrs

Figure S5

Coronal view sections

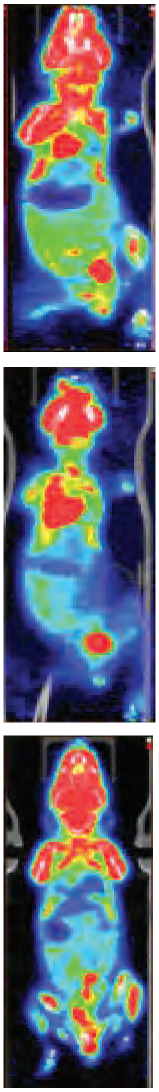

PRL (+)

Figure S6

A)

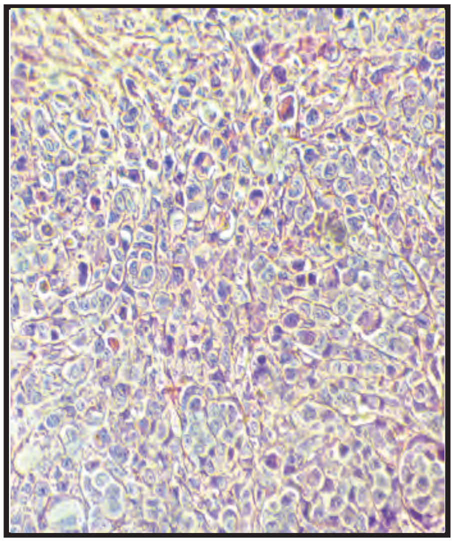

Primary Tumor 200X

B)

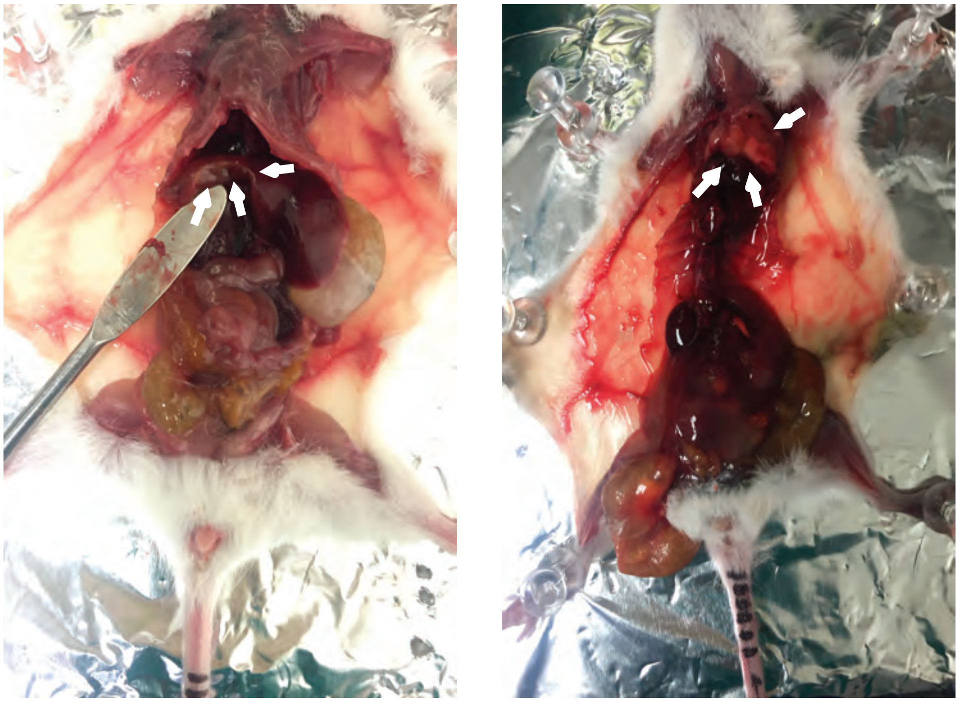

Liver

Thymus

C)

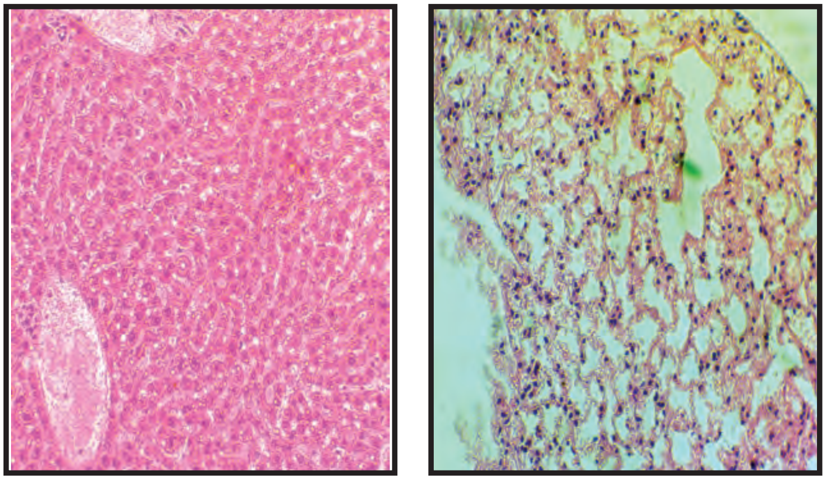

Liver 200X

Lung 200X

Figure S7

Supplementary Table-1

PRL (-)	Time after injection (hours)	Prolactin levels (ug/L)
1	2	<0.3
	4	<0.3
2	2	<0.3
	4	<0.3
3	2	<0.3
	4	<0.3

PRL (+)	Time after injection (hours)	Prolactin levels (ug/L)
1	2	53.2
	4	19.8
2	2	24.3
	4	22.7
3	2	0.9
	4	0.5
